# Comparison of Fecal Occult Blood Tests for Colorectal Cancer Screening in an Alaska Native Population With High Prevalence of *Helicobacter pylori* Infection, 2008–2012

**DOI:** 10.5888/pcd11.130281

**Published:** 2014-04-10

**Authors:** Diana Redwood, Ellen Provost, Elvin Asay, Diana Roberts, Donald Haverkamp, David Perdue, Michael G. Bruce, Frank Sacco, David Espey

**Affiliations:** Author Affiliations: Ellen Provost, Elvin Asay, Frank Sacco, Alaska Native Tribal Health Consortium, Anchorage, Alaska; Diana Roberts, Donald Haverkamp, David Espey, Centers for Disease Control and Prevention, Albuquerque, New Mexico; David Perdue, American Indian Cancer Foundation and Minnesota Gastroenterology PA, Minneapolis, Minnesota; Michael G. Bruce, Centers for Disease Control and Prevention, Anchorage, Alaska.

## Abstract

**Introduction:**

Alaska Native colorectal cancer (CRC) incidence and mortality rates are the highest of any ethnic/racial group in the United States. CRC screening using guaiac-based fecal occult blood tests (gFOBT) are not recommended for Alaska Native people because of false-positive results associated with a high prevalence of *Helicobacter pylori*-associated hemorrhagic gastritis. This study evaluated whether the newer immunochemical FOBT (iFOBT) resulted in a lower false-positive rate and higher specificity for detecting advanced colorectal neoplasia than gFOBT in a population with elevated prevalence of *H. pylori* infection.

**Methods:**

We used a population-based sample of 304 asymptomatic Alaska Native adults aged 40 years or older undergoing screening or surveillance colonoscopy (April 2008–January 2012).

**Results:**

Specificity differed significantly (*P* < .001) between gFOBT (76%; 95% CI, 71%–81%) and iFOBT (92%; 95% CI, 89%–96%). Among *H. pylori*-positive participants (54%), specificity of iFOBT was even higher (93% vs 69%). Overall, sensitivity did not differ significantly (*P* = .73) between gFOBT (29%) and iFOBT (36%). Positive predictive value was 11% for gFOBT and 32% for iFOBT.

**Conclusion:**

The iFOBT had a significantly higher specificity than gFOBT, especially in participants with current *H. pylori* infection. The iFOBT represents a potential strategy for expanding CRC screening among Alaska Native and other populations with elevated prevalence of *H. pylori*, especially where access to screening endoscopy is limited.

## Introduction

The incidence and mortality of colorectal cancer (CRC) among Alaska Native people is the highest of any ethnic or racial group in the United States (1). CRC is the leading incident cancer in Alaska Native people, who have nearly twice the incidence and mortality attributable to CRC as the general US population (2,3).

CRC can be treated more effectively if detected early using screening tests or even prevented through removal of precancerous lesions (4). CRC screening options include using a fecal occult blood test (FOBT), which detects blood in the stool resulting from CRC. Guaiac-based FOBT (gFOBT) detects the heme portion of hemoglobin. However, bleeding from upper intestinal tract lesions, including erosions, ulcers, and hemorrhagic gastritis from *Helicobacter pylori* infection (5) or nonsteroidal anti-inflammatory medications (6) may cause gFOBT false-positive results, as can nonhuman heme from ingesting red meat or ingesting foods with peroxidase activity (eg, spinach). Ingestion of vitamin C may cause false-negative tests. Dietary and medication restrictions are necessary for accurate gFOBTs. CRC screening using gFOBT has been discouraged among Alaska Native people as outlined in the CRC screening guidelines of the Alaska Area Native Health Service of June 2008. A high prevalence of *H. pylori* infection, which affects up to 75% of rural Alaska Native people (7), and the Alaska Native diet, which tends to be high in red meat (8), might contribute to false-positive gFOBTs and cause concern as to the overall reliability of the gFOBT in the Alaska Native population.

The immunochemical FOBT (iFOBT) detects the globin portion of human hemoglobin. Because globin is degraded as it transits the upper intestinal tract, iFOBT is used to detect lower intestinal bleeding. Dietary and medication restrictions are not required for iFOBT. For these reasons iFOBT has better specificity and equal or better sensitivity than gFOBT for the detection of colorectal neoplasms (9,10).

The purpose of this study was to evaluate whether iFOBT resulted in a lower false-positive rate and higher specificity than gFOBT for CRC screening in an Alaska Native population with elevated prevalence of *H. pylori* infection. Findings from this study may provide new evidence for use of iFOBT as a suitable alternative method of CRC screening for Alaska Native people and may have relevance for CRC screening in other populations with high *H. pylori* infection rates.

## Methods

### Study design

From April 2008 through January 2012 the study recruited from 2,340 patients scheduled for screening or surveillance colonscopy at the Alaska Native Medical Center in Anchorage, Alaska (Figure). Eligibility criteria included Alaska Native; 40 years old or older; no history of CRC or inflammatory bowel disease (eg, ulcerative colitis, Crohn’s disease); and no family history of familial adenomatous polyposis (FAP) or hereditary nonpolyposis colorectal cancer. Exclusion criteria included anticoagulant use that could not be discontinued during the study; frank blood in the stool in the previous 4 weeks; or residence in a home without a flush toilet (stool tests required toilet bowl water for sample collection). We excluded from the analysis participants who chose not to proceed with colonoscopic follow-up. Of the 700 total eligible adults, 397 (57%) were enrolled in the study. Of those, 304 (77%) completed the study.

**Figure:**
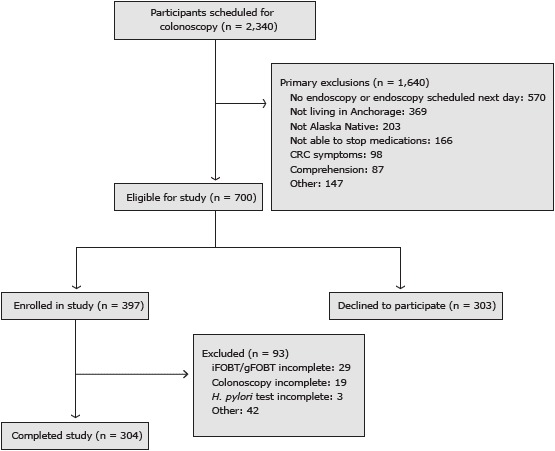
Flow diagram for enrollment in a study of fecal occult blood tests among Alaska Natives, Anchorage, Alaska, 2008–2012. Abbreviations: CRC, colorectal cancer; iFOBT, immunochemical fecal occult blood test; gFOBT, guaiac-based fecal occult blood test; *H. pylori, Helicobacter plyori*

The Alaska Area Institutional Review Board (IRB), the Indian Health Service IRB, and relevant tribal review committees approved the study protocol. The Centers for Disease Control and Prevention (CDC) Human Research Protection Office granted a reliance on the Alaska Area IRB for approval and oversight. Participants signed an informed consent before study enrollment.

### Study procedures

All participants completed an intake questionnaire for the collection of demographic, CRC risk factor, and medical and family history information. Participants were asked about living outside of the United States to assess potential international exposure to *H. pylori*. We gave each participant a noninvasive 13C-urea breath test (UBT; BreathTek Otsuka America Pharmaceutical, Inc, Lafayette, Colorado; www.breathtek.com) to determine current *H. pylori* infection status. As per clinical practice standards at the Alaska Native Medical Center, we informed participants with positive UBT results that, unless they had symptoms, treatment for *H. pylori* infection was not recommended. We asked persons with a negative UBT who were taking proton-pump inhibitors (PPIs) or bismuth-containing medication at study enrollment to retest after discontinuing PPIs for a minimum of 7 days to rule out false-negative UBTs.

We asked each participant within 7 days preceding the colonoscopy, but before starting the bowel preparation for the colonoscopy procedure, to complete a 3-card gFOBT (Hemoccult, Beckman Coulter, Fullerton, California) as well as a 2-card iFOBT (InSure FIT, Enterix, Edison, New Jersey) as per manufacturer directions. To maximize accuracy of gFOBT results, we instructed participants to avoid red meats and vitamin C in excess of 250 mg/d from supplements, citrus fruit, and juices for 3 days before and during stool collection. We also instructed participants to avoid nonsteroidal anti-inflammatory medicines such as ibuprofen, naproxen, or aspirin (more than 1 adult aspirin per day) for 7 days before and during stool collection. Patients gathered samples from 3 subsequent days of bowel movements (BMs). They took 2 samples from each of the 3 BMs for the gFOBT and 1 sample from 2 BMs for the iFOBT. Participants obtained stool samples for the gFOBT using a dry plastic container placed over the toilet bowl. Participants collected a sample of the stool using a specimen collection stick and spread the sample on the gFOBT card. They then transferred the stool to the toilet water where they collected the iFOBT samples by brushing the surface of the stool in the toilet water and applying the sample to the test card. They returned FOBTs to the study coordinators within 10 days of collection. Participants then had a screening or surveillance colonoscopy performed under conscious sedation by an endoscopist at the Alaska Native Medical Center. At the conclusion of all tests, participants completed a questionnaire assessing test instruction compliance and received a $75 gift card for their participation.

### Interpretation of study tests

Study research nurses analyzed the gFOBT cards according to manufacturer specifications. Quest Diagnostics, Inc, in Seattle, Washington, analyzed the iFOBT cards. As per standard practice, if any window on the 6-sample gFOBT or 2-sample iFOBT was positive, we considered that test positive. The 3 endoscopists and all pathologists were blinded to the FOBT results. CDC’s Arctic Investigations Program in Anchorage analyzed the *H. pylori* UBTs. Following colonoscopy, we classified participants on the basis of the most advanced lesion identified. To ensure quality control of data input, we randomly selected 20% of study records for validation with the original data sources (pathology reports, chart review, CDC laboratory, Quest Diagnostics, Inc).

### Outcome measures and statistical analysis

We determined a projected sample size of 300 using the McNemar test procedure for matched pairs with 80% power and an α of .05. The primary interest was the difference in specificity between gFOBT and iFOBT, which we estimated to be between 8% and 11%. We estimated the negativity rate for advanced colorectal neoplasia at 90%. Advanced colorectal neoplasia included invasive carcinoma, cancer in situ, adenomas with villous or tubulovillous histology, or tubular adenomas 1 cm or larger.

We performed the statistical analysis with SAS software (version 9.2, SAS Institute Inc, Cary, North Carolina). We calculated proportions and confidence intervals (CIs) for categorical data. We used binomial and Fisher exact test to calculate CIs for the specificity (and sensitivity) of the iFOBT and gFOBT using the results of the colonoscopy as the gold standard. We used Trajman’s method for McNemar test to compare specificity (and sensitivity) between the iFOBT and gFOBT (11). We created CIs for the difference in specificity (and sensitivity) of the 2 tests using the method described by Hawass (12).

## Results

The mean participant age was 56 years (62% female) (Table 1). The study included 156 participants (51%) who received a screening colonoscopy and 148 participants (49%) who received a surveillance colonoscopy. Eighty-eight participants (29%) had a family history of colorectal neoplasia, and 102 (34%) had a personal history of colorectal neoplasia.

**Table 1:** Demographics and Clinical Characteristics of Alaska Native People in Fecal Occult Blood Test (FOBT) Study, 2008–2012

Characteristic	No. (%) of Participants
**Total**	304 (100)
**Sex**	
Male	116 (38)
Female	188 (62)
**Age, y**	
40–49	33 (11)
50–64	230 (76)
≥65	41 (13)
**Reason for colonoscopy^a^**	
Screening	156 (51)
Past history of colorectal neoplasia	102 (34)
Family history of colorectal neoplasia^b^	88 (29)
**Medical history^c^**	
Stomach ulcers	34 (11)
Gastritis	41 (14)
Stomach bleeding	19 (6)
Heartburn or gastroesophageal reflux disease	131 (43)
Previous *Helicobactor pylori* infection	25 (8)
**Ever lived outside of the United States >6 months**	35 (12)
**Current medications**	
Antibiotics in last 14 days	20 (7)
Use of proton-pump inhibitors	44 (14)
Use of bismuth-containing medications	19 (6)
Use of vitamin C	123 (40)
Use of aspirin	117 (38)
Use of nonsteroidal anti-inflammatory medications	142 (47)
**Personal habits**	
Currently drink alcohol (mean alcoholic drinks per week = 7)	113 (37)
Ever smoked cigarettes	244 (80)
Currently smokes cigarettes (mean cigarettes per day = 9)	104 (34)
**Ever used home stool test before**	44 (14)
**Test instruction adherence**	
Avoided aspirin and nonsteroidal anti-inflammatory medications	217 (71)
Avoided red meat	172 (57)
Avoided vitamin C, fruit, and fruit juices	276 (91)
**Colonoscopy histology**	
No pathology reported	130 (43)
Normal biopsy	24 (8)
Hyperplastic	51 (17)
Adenoma, tubular <1 cm	66 (22)
Adenoma, tubular ≥1 cm^d^	12 (4)
Adenoma, tubulovillous^d^	10 (3)
Adenoma, villous^d^	1 (0)
Adenoma, serrated >5 mm^d^	1 (0)
Cancer in situ^d^	0
Adenocarcinoma^d^	4 (1)
Carcinoid	1 (0)
Other	4 (1)
***Helicobacter pylori* positive**	164 (54)

a Results do not add to 100% as participants could have more than 1 reason for colonoscopy.

b Includes cancer in a first-degree relative only.

c Includes history of self-reported risk factors that could be associated with a guaiac-based FOBT that was false positive.

d Considered to be advanced neoplasia, defined as any of the following: 1) adenomas ≥1 cm, 2) high-grade dysplasia, 3) villous histology, or 4) carcinoma.

Self-reported medical history that could be associated with a false-positive gFOBT included stomach ulcers (11%), gastritis (14%), stomach bleeding (6%), heartburn or gastroesophageal reflux disease (43%), and previous *H. pylori* infection (8%). Only 12% had ever lived outside of the United States for more than 6 months. Most participants were able to follow study restrictions to avoid aspirin and nonsteroidal anti-inflammatory medications (71%) and foods and juices containing vitamin C (91%). However, only 57% were able to follow the red meat restrictions. Of the 304 participants, 28 (9%) had advanced neoplasia. Of those with advanced neoplasia, 12 (43%) had tubular adenomas 1 cm or larger, 12 (43%) had tubulovillous, villous, or serrated adenomas, and 4 (14%) had adenocarcinomas. A total of 164 participants (54%) were positive for *H. pylori* by UBT.

There was a nonsignificant 7 percentage-point difference in overall sensitivity between gFOBT and iFOBT (*P* = .73, 95% CI, −13 to 27). For participants who tested positive for *H. pylori*, the difference in sensitivity was also nonsignificant at 4 percentage points (*P =* >.99, 95% CI, −20 to 29). Overall specificity for advanced neoplasia was 76% (95% CI, 71%–81%) for gFOBT and 92% (95% CI, 89%−96%) for iFOBT, a significant difference of 16 percentage points (*P* < .001, 95% CI, 10–22) (Table 2). Specificity did not differ significantly by sex or age. For *H. pylori*-positive participants, the difference in specificity (24 percentage points) between the 2 tests was even greater (*P* < .001, 95% CI, 17−31). Among participants who were able to adhere to dietary and medication restrictions, specificity of gFOBT increased but was still significantly lower than iFOBT. Of the 4 patients identified with adenocarcinoma, 2 were identified by gFOBT and 1 by iFOBT.

**Table 2:** Sensitivity and Specificity of Guaiac-Based Fecal Occult Blood Test (gFOBT) and Immunochemical Fecal Occult Blood Test (iFOBT) for Detection of Advanced Neoplasia^a^ Among Alaska Native People by *Helicobacter pylori* Infection Status, 2008–2012

*H. pylori* status/Test Used	Sensitivity, % (95% CI)	Specificity, % (95% CI)	PPV, % (95% CI)
**Total study population (n = 304)**			
gFOBT	29 (12–45)	76 (71–81)	11 (4–17)
iFOBT	36 (18–53)	92 (89–96)	32 (16–49)
***H. pylori*-positive (n = 164)**			
gFOBT	29 (9–48)	69 (62–77)	12 (3–21)
iFOBT	33 (13–54)	93 (89–97)	41 (18–65)
***H. pylori*-negative (n = 140)**			
gFOBT	29 (4–71)^b^	83 (76–89)	8 (1–26)^b^
iFOBT	43 (10–82)^b^	92 (86–96)	21 (5–51)^b^

Abbreviations: CI, confidence interval; PPV, positive predictive value.

a Advanced neoplasia defined as any of the following: 1) adenomas ≥1 cm, 2) high-grade dysplasia, 3) villous histology, or 4) carcinoma.

b Exact CI.

Among 276 participants without advanced neoplasia, 67 (24%) had a false-positive gFOBT; only 21 (8%) were false positives with iFOBT (Table 3). For *H. pylori*-positive participants without advanced neoplasia (n = 143), a total of 44 (31%) were falsely identified as positive by the gFOBT compared with 10 (7%) false positive by the iFOBT. For *H. pylori*-negative participants without advanced neoplasia (n = 133), 23 (17%) had a false positive gFOBT compared with 11 (8%) false positives by iFOBT. The PPV for gFOBT was 11% whereas the PPV for iFOBT was 32%. Likelihood ratios for gFOBT were close to 1 for the total population and for *H. pylori*-positive and *H. pylori*-negative populations. Likelihood ratios for the iFOBT were 4.6 for the total population, 4.8 for *H. pylori*-positive populations, and 4.6 for *H. pylori*-negative populations.

**Table 3:** Outcomes of Stool Tests for Colorectal Cancer Screening for Detection of Advanced Neoplasia^a^ Among Alaska Natives by *Helicobacter pylori *Status (n = 304), 2008–2012

Test Results	*H. pylori* Positive		*H. pylori* Negative		Total	
	Advanced Neoplasia Positive, n (%)	Advanced Neoplasia Negative, n (%)	Advanced Neoplasia Positive, n (%)	Advanced Neoplasia Negative, n (%)	Advanced Neoplasia Positive, n (%)	Advanced Neoplasia Negative, n (%)
**Immunochemical fecal occult blood test**						
Positive	7 (33)	10 (7)	3 (43)	11 (8)	10 (36)	21 (8)
Negative	14 (67)	133 (93)	4 (57)	122 (92)	18 (64)	255 (92)
**Guaiac-based fecal occult blood test**						
Positive	6 (29)	44 (31)	2 (29)	23 (17)	8 (29)	67 (24)
Negative	15 (71)	99 (69)	5 (71)	110 (83)	20 (71)	209 (76)

a Advanced neoplasia defined as any of the following: 1) adenomas ≥1 cm, 2) high-grade dysplasia, 3) villous histology, or 4) carcinoma.

## Discussion

There is widespread reluctance to use gFOBT for CRC screening in Alaska Native people because of a concern that the high prevalence of *H. pylori* infection and other factors lead to excessive false-positive tests and poor specificity. Haverkamp et al found that Alaska had the lowest percentage (54%) of tribal providers nationally that reported using FOBT for CRC screening (13). This study corroborates these concerns and indicates a substantial gain in specificity when using iFOBT compared with gFOBT. The advantages of iFOBT compared with gFOBT were most evident in participants with *H. pylori* infection. Other studies have reported specificities of gFOBT for detecting advanced neoplasia from 63% to 97% and of iFOBT from 60% to 99% (10,14–19). Test specificity in this study population fell within the previously reported upper range for iFOBT. Sensitivity for both tests was consistent with previous reports. The false-positive rate for both iFOBT (8%) and gFOBT (24%) was lower than previously reported (48% to 87%) in other studies, including a study in a Taiwanese population with a high prevalence of *H. pylori* infection, which showed an iFOBT false-positive rate of 59% (20).

As Alaska Native people have the highest CRC incidence and mortality of any US population, accessible and reliable CRC screening is a critical public health objective. Yet the reluctance to use FOBTs has led to a reliance on endoscopy, particularly colonoscopy, as the preferred CRC screening method in this population (21). Compared with stool tests, endoscopy requires specially trained providers, is resource intensive, and requires patients to undergo invasive bowel preparation and screening procedures. Furthermore, as elsewhere in the United States (22), endoscopic capacity is limited in Alaska, particularly at the regional health facilities that serve the Alaska Native population. For many Alaska Native people living in remote communities, obtaining screening endoscopy requires costly and time-consuming air travel to 1 of the 7 regional health facilities with endoscopic capacity or to Anchorage. Because of a shortage of trained local personnel, only 4 of these regional facilities provide endoscopy on a continuous basis, while 3 hold intermittent screening clinics staffed by itinerant endoscopists. Because of these geographic and health system barriers, there is a need to expand CRC screening options such as FOBT for Alaska Native people, particularly for those living in rural regions of the state.

Human infection with *H. pylori* is common (23). Our study of mostly urban Alaska Native adults found that 54% of participants were *H. pylori*-positive by UBT, which is higher than the US average seropositivity rate (31%) (24). These data may have relevance for other settings because of the high prevalence of *H. pylori* infection worldwide, especially in low-resource countries with less access to screening endoscopy and greater need of low-cost screening methods like stool testing.

The dietary modification required for gFOBT is also a challenge in the Alaska Native population. Study participants showed a high level of nonadherence to the gFOBT dietary restrictions, especially to red meat avoidance. Other studies have examined FOBT completion rates and dietary restriction (25,26), but none have assessed adherence by Alaska Native people to FOBT dietary restriction. Alaska Native people have historically lived entirely on foods hunted and gathered from the land and sea (traditional foods). Most Alaska Native diets were heavily meat- and fat-based, with few carbohydrates (8). Although store-bought foods have increasingly supplemented or supplanted traditional foods, Alaska Native people still acquire a high percentage of their protein and associated micronutrients from wild foods (27). This is especially so among Alaska Native elders, who tend to have a higher intake of traditional foods than their younger counterparts, including more fish, moose, seal, and walrus consumption (28). These traditional diet patterns, along with the higher expense of store-bought foods, may help to explain the apparent reluctance of study participants to avoid red meat as required for the gFOBT.

These data suggest that iFOBT offers advantages over gFOBT. Screening rates across the Alaska Tribal Health System (ATHS) are lower than rates among US whites, with 58% of age-appropriate Alaska Native people being up to date with CRC screening in 2012 (ATHS data, 2000–2012) (29). The use of iFOBT in other populations to increase adherence to CRC screening recommendations (30,31) may help increase CRC screening rates in Alaska Native people while allowing for prioritization of colonoscopy for diagnostic and surveillance examinations. The iFOBT is simple, convenient, relatively inexpensive, and does not require dietary or drug restrictions. However, as shown in our results, 6% of participants had a false-negative iFOBT, and the iFOBT only correctly identified 1 of the 4 adenocarcinomas, which emphasizes the need for patient education to ensure annual iFOBT adherence to detect cancers that might be missed in the previous screening test cycle. Additionally, expanding screening options to include iFOBT would require multiple changes within the ATHS. These include system-wide policy changes approved by multiple tribal health care providers and tribal leadership; development of new health education materials for both patients and providers; systems changes to enable local laboratory processing of the iFOBT kits; and coordination of appropriate follow-up for positive results with diagnostic colonoscopy. A pilot intervention in 2 Alaskan regions is being planned to evaluate the feasibility and acceptability of iFOBT in the Alaska Native population, especially among persons who have previously refused screening by endoscopy. This pilot project aims to lay the groundwork for broader implementation of the use of iFOBT throughout the ATHS.

The comparison of iFOBT to gFOBT study findings reported here are subject to several limitations. The small sample size limited the ability to stratify analysis by age group, sex, or subgroups such as those with advanced CRC neoplasia. Although the prevalence of advanced neoplasia among participants was high (9%), the absolute number of cases widened confidence intervals around the estimated sensitivity of both tests. The gFOBT was a 6-sample test, compared with the 2-sample iFOBT, which might have increased the potential for false-positive gFOBT compared with the iFOBT results. In addition, gFOBT and iFOBT are designed to screen for cancer, not advanced adenomas, which often do not cause fecal blood loss. Nonetheless, the differences demonstrated between the 2 tests in this population, especially among those with *H. pylori* infection as measured by UBT, show a higher comparative effectiveness of iFOBT to gFOBT. Lastly, this study only included Alaska Native adults living in an urban area of Alaska and may not reflect results of a similar study in Alaska Native residents in communities where *H. pylori* prevalence may be higher.

This study found that iFOBT had a significantly higher specificity than gFOBT, especially in participants with *H. pylori* infection. The data provide strong evidence that would support a decision to offer iFOBT as an alternative for CRC screening in rural and remote populations with a limited capacity for screening colonoscopy. Furthermore, these data have relevance to the worldwide CRC screening of populations with high prevalence of *H. pylori* infection, many of which are also low-resource and have a greater need of low-cost screening methods like stool testing. Increasing the availability and types of tests for CRC screening may increase uptake of screening and may ultimately decrease the excess illness and death associated with this disease among Alaska Native people.

## References

[1] Espey DK , Wu XC , Swan J , Wiggins C , Jim MA , Ward E , Annual report to the nation on the status of cancer, 1975–2004, featuring cancer in American Indians and Alaska Natives. Cancer 2007;110(10):2119–52. 10.1002/cncr.23044 17939129

[2] Surveillance, Epidemiology, and End Results (SEER)*Stat Database: Mortality - All COD, Aggregated With State, Total U.S. (1990–2008) <Katrina/Rita Population Adjustment>. National Cancer Institute, DCCPS, Surveillance Research Program, Cancer Statistics Branch. 2011. http://www.seer.cancer.gov. Accessed August 13, 2012.

[3] Kelly JJ , Alberts SR , Sacco F , Lanier AP . Colorectal cancer in Alaska Native people, 2005–2009. Gastrointest Cancer Res 2012;5(5):149–54. 23112882 PMC3481146

[4] Levin B , Lieberman DA , McFarland B , Smith RA , Brooks D , Andrews KS , Screening and surveillance for the early detection of colorectal cancer and adenomatous polyps, 2008: a joint guideline from the American Cancer Society, the US Multi-Society Task Force on Colorectal Cancer, and the American College of Radiology. CA Cancer J Clin 2008;58(3):130–60. 10.3322/CA.2007.0018 18322143

[5] Kusters JG , van Vliet AH , Kuipers EJ . Pathogenesis of *Helicobacter pylori* infection. Clin Microbiol Rev 2006;19(3):449–90. 10.1128/CMR.00054-05 16847081 PMC1539101

[6] Yip R , Limburg PJ , Ahlquist DA , Carpenter HA , O’Neill A , Kruse D , Pervasive occult gastrointestinal bleeding in an Alaska native population with prevalent iron deficiency. Role of *Helicobacter pylori* gastritis. JAMA 1997;277(14):1135–9. 10.1001/jama.1997.03540380049030 9087468

[7] Parkinson AJ , Gold BD , Bulkow L , Wainwright RB , Swaminathan B , Khanna B , High prevalence of *Helicobacter pylori* in the Alaska native population and association with low serum ferritin levels in young adults. Clin Diagn Lab Immunol 2000;7(6):885–8. 11063492 10.1128/cdli.7.6.885-888.2000 PMC95979

[8] Ballew C , Ross Tzilkowski A , Hamrick K , Nobmann ED . The contribution of subsistence foods to the total diet of Alaska Natives in 13 rural communities. Ecol Food Nutr 2006;45(1):1–26. 10.1080/03670240500408302

[9] Levi Z , Hazazi R , Rozen P , Vilkin A , Waked A , Niv Y . A quantitative immunochemical faecal occult blood test is more efficient for detecting significant colorectal neoplasia than a sensitive guaiac test. Aliment Pharmacol Ther 2006;23(9):1359–64. Erratum in Aliment Pharmacol Ther 2006;24(5):895. 10.1111/j.1365-2036.2006.02898.x 16629942

[10] Smith A , Young GP , Cole SR , Bampton P . Comparison of a brush-sampling fecal immunochemical test for hemoglobin with a sensitive guaiac-based fecal occult blood test in detection of colorectal neoplasia. Cancer 2006;107(9):2152–9. 10.1002/cncr.22230 16998938

[11] Trajman A , Luiz RR . McNemar chi2 test revisited: comparing sensitivity and specificity of diagnostic examinations. Scand J Clin Lab Invest 2008;68(1):77–80. 18224558 10.1080/00365510701666031

[12] Hawass NE . Comparing the sensitivities and specificities of two diagnostic procedures performed on the same group of patients. Br J Radiol 1997;70(832):360–6. 9166071 10.1259/bjr.70.832.9166071

[13] Haverkamp D , Perdue DG , Espey D , Cobb N . A survey of Indian Health Service and tribal health providers’ colorectal cancer screening knowledge, perceptions, and practices. J Health Care Poor Underserved 2011;22(1):243–57. 21317519 10.1353/hpu.2011.0014

[14] Cheng TI , Wong JM , Hong CF , Cheng SH , Cheng TJ , Shieh MJ , Colorectal cancer screening in asymptomaic adults: comparison of colonoscopy, sigmoidoscopy and fecal occult blood tests. J Formos Med Assoc 2002;101(10):685–90. 12517041

[15] Greenberg PD , Bertario L , Gnauck R , Kronborg O , Hardcastle JD , Epstein MS , A prospective multicenter evaluation of new fecal occult blood tests in patients undergoing colonoscopy. Am J Gastroenterol 2000;95(5):1331–8. 10.1111/j.1572-0241.2000.02032.x 10811348

[16] Hoepffner N , Shastri YM , Hanisch E , Rösch W , Mössner J , Caspary WF , Comparative evaluation of a new bedside faecal occult blood test in a prospective multicentre study. Aliment Pharmacol Ther 2006;23(1):145–54. 10.1111/j.1365-2036.2006.02702.x 16393292

[17] Nakama H , Fattah A , Zhang B , Uehara Y , Wang C . A comparative study of immunochemical fecal tests for detection of colorectal adenomatous polyps. Hepatogastroenterology 2000;47(32):386–9. 10791196

[18] Rozen P , Knaani J , Papo N . Evaluation and comparison of an immunochemical and a guaiac faecal occult blood screening test for colorectal neoplasia. Eur J Cancer Prev 1995;4(6):475–81. 10.1097/00008469-199512000-00006 8580783

[19] Young GP , St John DJ , Cole SR , Bielecki BE , Pizzey C , Sinatra MA , Prescreening evaluation of a brush-based faecal immunochemical test for haemoglobin. J Med Screen 2003;10(3):123–8. 10.1258/096914103769011012 14561263

[20] Chiang TH , Lee YC , Tu CH , Chiu HM , Wu MS . Performance of the immunochemical fecal occult blood test in predicting lesions in the lower gastrointestinal tract. CMAJ 2011;183(13):1474–81. 10.1503/cmaj.101248 21810951 PMC3176840

[21] Redwood D , Provost E , Perdue D , Haverkamp D , Espey D . The last frontier: innovative efforts to reduce colorectal cancer disparities among the remote Alaska Native population. Gastrointest Endosc 2012;75(3):474–80. 10.1016/j.gie.2011.12.031 22341095 PMC4523058

[22] Seeff LC , Manninen DL , Dong FB , Chattopadhyay SK , Nadel MR , Tangka FK , Is there endoscopic capacity to provide colorectal cancer screening to the unscreened population in the United States? Gastroenterology 2004;127(6):1661–9. 10.1053/j.gastro.2004.09.052 15578502

[23] Brown LM . *Helicobacter pylori*: epidemiology and routes of transmission. Epidemiol Rev 2000;22(2):283–97. 10.1093/oxfordjournals.epirev.a018040 11218379

[24] Grad YH , Lipsitch M , Aiello AE . Secular trends in *Helicobacter pylori* seroprevalence in adults in the United States: evidence for sustained race/ethnic disparities. Am J Epidemiol 2012;175(1):54–9. 10.1093/aje/kwr288 22085628 PMC3244610

[25] Pignone M , Campbell MK , Carr C , Phillips C . Meta-analysis of dietary restriction during fecal occult blood testing. Effective clinical practice. Err Clin Pract 2001;4(4):150–6. 11525101

[26] Joseph A . Compliance with fecal occult blood testing: the role of restrictive diets. Am J Public Health 1988;78(7):839–41. 10.2105/AJPH.78.7.839 3381964 PMC1350349

[27] Bersamin A , Zidenberg-Cherr S , Stern JS , Luick BR . Nutrient intakes are associated with adherence to a traditional diet among Yup’ik Eskimos living in remote Alaska Native communities: the CANHR Study. Int J Circumpolar Health 2007;66(1):62–70. 10.3402/ijch.v66i1.18228 17451135

[28] Redwood DG , Ferucci ED , Schumacher MC , Johnson JS , Lanier AP , Helzer LJ , Traditional foods and physical activity patterns and associations with cultural factors in a diverse Alaska Native population. Int J Circumpolar Health 2008;67(4):335–48. 10.3402/ijch.v67i4.18346 19024803 PMC2925499

[29] Indian Health Service. Alaska area aggregate GPRA [Government Performance and Results Act of 1993] clinical performance report, CRS [Clinical Reporting System] Version 10.0. Anchorage (AK): Alaska Area Native Health Service; 2012.

[30] Kluhsman BC , Lengerich EJ , Fleisher L , Paskett ED , Miller-Halegoua SM , Balshem A , A pilot study for using fecal immunochemical testing to increase colorectal cancer screening in Appalachia, 2008–2009. Prev Chronic Dis 2012;9:E77. 22482136 PMC3392085

[31] Quintero E , Castells A , Bujanda L , Cubiella J , Salas D , Lanas Á , Colonoscopy versus fecal immunochemical testing in colorectal-cancer screening. N Engl J Med 2012;366(8):697–706. 10.1056/NEJMoa1108895 22356323

